# The Feed Additive Potassium Diformate Prevents *Salmonella enterica Serovar Pullorum* Infection and Affects Intestinal Flora in Chickens

**DOI:** 10.3390/antibiotics11091265

**Published:** 2022-09-18

**Authors:** Yufan Sun, Panyuan Yu, Yiluo Cheng, Jiahui Liu, Xiabing Chen, Tengfei Zhang, Ting Gao, Rui Zhou, Lu Li

**Affiliations:** 1 State Key Laboratory of Agricultural Microbiology, The Cooperative Innovation Center for Sustainable Pig Production Co-sponsored by Province and Ministry, College of Veterinary Medicine, Huazhong Agricultural University, Wuhan 430070, China; 2 Institute of Animal Husbandry and Veterinary Science, Wuhan Academy of Agricultural Sciences, Wuhan 430208, China; 3 Key Laboratory of Prevention and Control Agents for Animal Bacteriosis (Ministry of Agriculture and Rural Affairs), Hubei Provincial Key Laboratory of Animal Pathogenic Microbiology, Institute of Animal Husbandry and Veterinary, Hubei Academy of Agricultural Sciences, Wuhan 430064, China; 4 International Research Center for Animal Disease, Ministry of Science and Technology of China, Wuhan 430070, China

**Keywords:** potassium diformate, *Salmonella pullorum*, intestinal flora, chickens

## Abstract

Extensive studies have shown that potassium diformate (KDF), an antibiotic substitute used as a feed additive, improves animal growth performance, although there is less direct evidence of its preventive effect on bacterial infections and its influence on the intestinal flora of animals. In this study, the inhibition effect of KDF on *Salmonella enterica serovar Pullorum*, an important enteric pathogen causing pullorum disease, was investigated in vitro and on a chicken infection model. The effect of KDF on the diversities and structures of chicken duodenal and cecum flora were also investigated using 16S rRNA gene sequencing. The results showed that addition of 0.5% KDF in feed or 0.1% KDF in drinking water significantly reduced the bacterial loads and the degree of pathological changes in the cecum, improved digestion and reduced the pH of the gastrointestinal tract of chickens infected with *S. pullorum*. KDF also significantly modified the diversity and abundance of intestinal microflorae in chickens. In particular, it promoted the colonization of several probiotics, such as *Bacteroides*, *Blautia*, *Ruminococcus_torques_group* and *Faecalibacteriumm*, which are involved in maintenance of the intestinal barrier, modulation of inflammation, energy supply for intestinal cells and pathogen resistance. These results enrich the theoretical basis for the clinical application of KDF in chickens.

## 1. Introduction

Many regulations have been made concerning human and animal health to limit the use of antibiotics globally [1]. Consequently, the application of substitutes for antibiotics that improve animal health and welfare is particularly important. Organic acid has been applied in the animal breeding industry for more than five decades [2]. Potassium diformate (KDF, HCOOH·HCOOK; molecular weight, 130.14 g/mol), a type of organic acid, is a white crystalline compound consisting of formic acid (FA) and potassium formate linked via hydrogen and covalent bonds that is highly hygroscopic and easily soluble in water, with low volatility and no undesirable odor. Compared with formate, it is less corrosive. It is dissociated into formic acid and potassium ions in the stomach after being ingested by animals. It enters the intestine with food, effectively solving the problem of the inability of formic acid to reach the intestine. KDF is the first substitute approved as a non-antibiotic growth promoter by the European Union (Commission Reg (EC) number 1334/2001) [3]. In 2005, the Ministry of Agriculture and Rural Affairs of the People’s Republic of China approved KDF as a new feed additive to be used in livestock.

Dietary KDF has demonstrated effectiveness in enhancing growth performance and nutrient utilization in animals. In weaning piglets, dietary supplementation with KDF improved weight gain, feed intake and feed conversion [4,5]. KDF in broiler feeds has positive effects on performance, immune parameters [6]. Many studies have shown that dietary KDF improves growth performance of different varieties of fish, enhances the activity of digestive enzymes and stimulates the proliferation of intestinal probiotics [7,8,9]. Gastric pH tends to be high due to insufficient secretion of endogenous HCl when the gastrointestinal tract (GIT) of newly weaned piglets is not fully developed. Organic acid supplementation helps to solve this problem [10]. Furthermore, KDF provides potassium ions, which can regulate the electrolyte balance and efficiently improve amino acid utilization [11].

On the other hand, organic acids also affect the activities of microbes. The recognized mechanism of bactericidal effects of organic acids is diffusion into the bacterial cell cytoplasm, dissociation inside the cell and release of H^+^ ions. To redress the imbalance, the bacterial cell is forced to use energy to expel protons across the membrane via the H^+^-ATPase pump. In addition, the anion RCOO^−^ is toxic to bacterial DNA replication, disrupting metabolic functions and increasing osmotic cell pressure [1]. KDF has been confirmed to exhibit antimicrobial activity in vitro. The minimum inhibitory concentration (MIC) of KDF in vitro against a variety of pig pathogens, including *Staphylococcus aureus*, *Streptococcus suis*, *Actinobacillus pleuropneumoniae* and *Salmonella typhimurium*, was reported as 1.95 mg·mL^−1^ [12]. In vivo, the addition of KDF to a starter diet for piglets decreased total anaerobic bacteria, lactic acid bacteria, coliforms and yeasts in feces and in digesta from various segments of the GIT [13]. In finishing pigs, the addition of KDF in water or feed is a useful strategy to reduce *Salmonella* prevalence on the farm [14,15,16]. The total bacteria per gram of feces was significantly reduced in fish fed a KDF diet [17].

Based on pathogen challenge assay, KDF has also shown promising effects with respect to resistance against intestinal disease. In weaning piglets, adding KDF to the diet resulted in significantly lower counts of bacteria in the GIT after *Salmonella Derby* or *Escherichia coli* infection, indicating a protection effect of KDF of the GIT barrier [18,19]. In broiler chickens, KDF reduced the number of *Clostridium perfringens* in the jejunum and mortality using the necrotic enteritis model [20]. In aquatic species, the mortality of *O. niloticus* fed on KDF and challenged with *Aeromonas hydrophila* or *Francisella noatunensis* subsp. *orientalis* were lower than that of the control group [21,22].

Infectious diseases still constitute a major constraint in the poultry industry. *S. pullorum* is a pathogen specific to birds that causes pullorum disease in young chickens, leading to considerable economic losses in the poultry industry [23]. In China, pullorum disease is in the eradication phase. Although KDF has been widely used in the poultry industry, whether it can help to prevent *S. pullorum* is still unknown. The effect of KDF supplementation on the community of intestinal flora of chickens is also unclear. Hence, in this study, we evaluated the preventive effect of KDF on *S. pullorum* using a chicken infection model. The influence of KDF on the intestinal flora of chickens was further analyzed.

## 2. Results

### 2.1. Effect of Supplementation of KDF in Diets or Water on the Resistance of Chickens to S. pullorum

**Figure 1:**
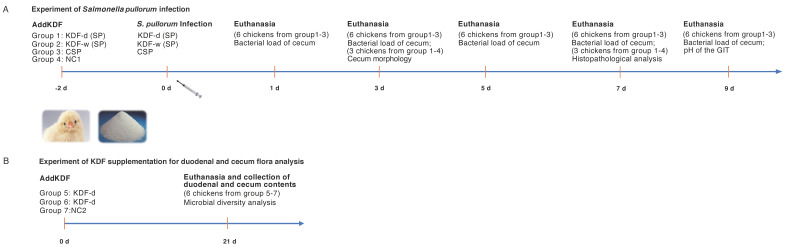
Animal experiment protocol. (**A**) Animal infection experiment protocol. KDF-d (SP): chickens supplied with 0.5% KDF in diet infected with *S. pullorum* (n = 30); KDF-w (SP): chickens supplied with 0.1% KDF in drinking water infected with *S. pullorum* (n = 30); CSP: chickens supplied with non-supplemented diet and water infected with *S. pullorum* (n = 30); NC1: chickens supplied with non-supplemented diet and water without infection (n = 10). (**B**) KDF supplementation experiment protocol for duodenal and cecum flora analysis. KDF-d: chickens supplied with 0.5% KDF in diet without infection (n = 6); KDF-w: chickens supplied with 0.1% KDF in drinking water without infection (n = 6); NC2: chickens supplied with non-supplemented diet and water without infection (n = 6).

**Figure 2:**
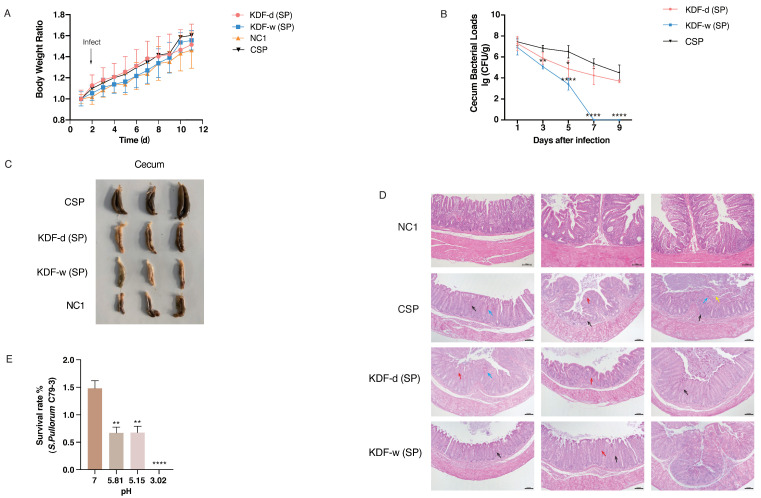
The addition of KDF reduces the susceptibility of chickens to *S. pullorum* infection. (**A**) Changes in body weight of the chickens. (**B**) *S. pullorum* loads in the cecum (n = 6). (**C**) Cecum morphology of chickens (n = 3). (**D**) Pathological changes in the cecum of chickens in different groups (n = 3). Black arrow: inflammatory cell infiltration; red arrow: decrease in goblet cells in the mucosal layer; blue arrow: congestion of the interstitial blood vessels; yellow arrow: mucosal epithelial cell necrosis and nuclei pyknosis, dissolution and disappearance. (**E**) Survival rate of *S. pullorum C79-3* at different pH values (n = 3). KDF-d (SP): chickens supplied with 0.5% KDF in diet infected with *S. pullorum*; KDF-w (SP): chickens supplied with 0.1% KDF in drinking water infected with *S. pullorum*; CSP: chickens supplied with non-supplemented diet and water infected with *S. pullorum*; NC1: chickens supplied with non-supplemented diet and water without infection. All data are shown as means ± SD. * *p* < 0.05, ** *p* < 0.01, **** *p* < 0.0001.

In this study, a chicken model was used to investigate the protective effect of KDF against *S. pullorum* infection (Figure 1A). No mortality by inoculation was observed during the experiment. There were no significant differences in the body weight of chickens between the non-supplemented group without infection (NC1), the non-supplemented group infected with *S. pullorum* (CSP), the group supplied with a 0.5% KDF in diet infected with *S. pullorum* (KDF-d (SP)) and the group supplied with 0.1% KDF in drinking water infected with *S. pullorum* (KDF-w (SP)) (Figure 2A). However, compared with the CSP group, chickens in the KDF-w (SP) group had remarkably lower bacterial loads in cecum of 3 dpi, and bacteria was totally cleared 7 days post infection (dpi) (Figure 2B). Furthermore, the bacterial loads in cecum of chickens in the KDF-d (SP) group were reduced significantly at 5 dpi compared with the CSP group (Figure 2B). A comparison of cecum morphology revealed that the chickens in the CSP group had increased cecum contents relative to the NC1 group. However, the chickens in the KDF-d/w (SP) groups had lower cecum contents than the other groups (Figure 2C). These results showed that KDF could effectively suppress the colonization of *S. pullorum* and alleviate the reduced digestive function caused by *S. pullorum* in the cecum of chickens. The effect of supplementation in drinking water was more effective than that by feeding. Additionally, histopathological analysis demonstrated that the overall structure of the cecum of the chickens in the CSP group was abnormal at 7 dpi, with dilation and congestion of blood vessels, inflammatory cell infiltration and notably reduced goblet cells (Figure 2D). Conversely, the cecum of chickens in the KDF-d/w (SP) groups showed nearly normal structural integrity, with only slight infiltration of inflammatory cells and a reduction in goblet cells (Figure 2D). The above results indicate that supplementation of KDF in feed or water can reduce the bacterial loads and pathological changes in the cecum of chickens and therefore effectively prevent *S. pullorum* infection.

### 2.2. Changes in the pH Conditions of the GIT

Chickens were euthanized at 9 dpi, and the pH values of different sections of the GIT were measured. Compared with the CSP group, the pH values of the duodenum, jejunum, ileum and cecum of chickens in the KDF-d(SP) group and gizzard, as well as all intestinal segments in the KDF-w (SP) group, were significantly reduced (Table 1). Hence, pretreatment with KDF reduces pH values in the GIT of chickens, with a more significant effect resulting from supplementation in water.

### 2.3. Antibacterial Activity of KDF In Vitro

Based on the pH of different sections of the GIT as measured in this study, selected pH values (3.02, 5.15 and 5.81) of the GIT (gizzard, duodenum and cecum) of chickens were simulated by dissolving different concentrations of KDF in ddH_2_O in vitro. Compared with that in a neutral environment, *S. pullorum* exhibited significantly reduced survivability in acidic environments (*p* < 0.01) (Figure 2E). The data demonstrated that KDF inhibits the proliferation of *S. pullorum* in a dose-dependent manner and is positively correlated with decreased pH values.

### 2.4. Microbial Diversity of the Duodenum and Cecum Microbiota

The duodenal and cecal contents of uninfected groups, including chickens supplied with 0.5% KDF in diet without infection (KDF-d), chickens supplied with 0.1% KDF in drinking water without infection (KDF-w), chickens supplied with non-supplemented diet and water without infection (NC2), were collected for 16S rDNA sequencing to analyze gut microbiota (Figure 1B). There were no significant differences in the body weight of chickens between the KDF-d, KDF-w and NC2 groups (Supplementary Materials Figure S1). In the duodenal flora, a total of 1,684,223 high-quality sequences were obtained, and 88,020 ± 17,096, 97,000 ± 30,668 and 95,684 ± 19,763 sequences were obtained from the KDF-d, KDF-w and NC2 groups, respectively (Supplementary Materials Table S1). Furthermore, these sequences were assigned to 744 OTUs based on 97% sequence similarity. A total of 309 OTUs shared by the three groups were identified, accounting for 41.53% of all sequences (Supplementary Materials Figure S2). In the cecal flora, a total of 1,884,791 high-quality sequences were obtained, and 76,613 ± 34,383, 135,877 ± 44,595 and 101,642 ± 19,297 sequences were obtained from the KDF-d, KDF-w and NC2 groups, respectively (Supplementary Materials Table S1). Then, these sequences were assigned to 1991 OTUs based on 97% sequence similarity. A total of 972 OTUs shared by the three groups were identified, accounting for 48.82% of all sequences (Supplementary Materials Figure S3).

**Figure 3:**
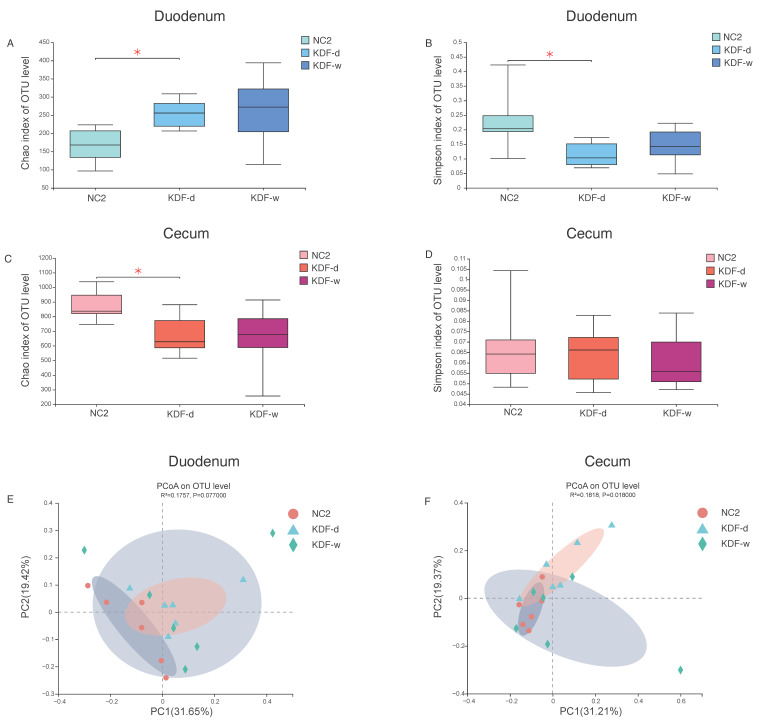
Gut microbial diversity in the investigated groups. Alpha diversity of the duodenum (**A**,**B**) and cecum (**C**,**D**) are determined by Chao1 index (**A**,**C**) and Simpson index (**B**,**D**), respectively. Beta diversity based on PCoA of the duodenum (**E**) and cecum (**F**) are also presented. NC2: chickens supplied with non-supplemented diet and water without infection (n = 6); KDF-d: chickens supplied with 0.5% KDF in diet without infection (n = 6); KDF-w: chickens supplied with 0.1% KDF in drinking water without infection (n = 6). * *p* < 0.05.

Effects on richness and diversity of the intestinal microbiota were estimated by the Chao 1 and Simpson index for α-diversity analysis. In the duodenal flora, as compared to the NC2 group, a significant increase in Chao was observed in the KDF-d group (*p* < 0.05), as well as a remarkable decrease in the Simpson index (*p* < 0.05) (Figure 3A,B). In the cecal flora, compared with the NC2 group, the KDF-d group exhibited a significant elevation in the Chao index (*p* < 0.05) and no significant changes in Simpson index (Figure 3C,D). There was no significant alteration in α-diversity in the KDF-w group in either intestinal segments. Principal coordinate analysis (PCoA) based on the weighted Bray–Curtis distance showed that samples from different groups were scattered to varying degrees but with no significant difference in duodenum (R^2^ = 0.1757, *p* = 0.0770) (Figure 3E). However, the KDF treatment dramatically influenced bacterial communities in cecum (R^2^ = 0.1818, *p* = 0.0180) (Figure 3F). The relationships in gut microbiota between different groups were calculated using permutational multivariate analysis of variance (PERMANOVA) based on Bray–Curtis distance and OTU level, further validated above results; both intestinal segments in the KDF-d group differed significantly from those of the NC2 group (*p* < 0.05). Notably, in the cecal flora, there was a remarkable change between the KDF-d and KDF-w groups (Table 2).

The supplementation of KDF in diets affects the microbial diversity of the duodenum and cecum of chickens, whereas water supplementation has no significant effect.

### 2.5. Effects of KDF Supplementation on Composition of the Duodenum and Cecum Microbiota

**Figure 4:**
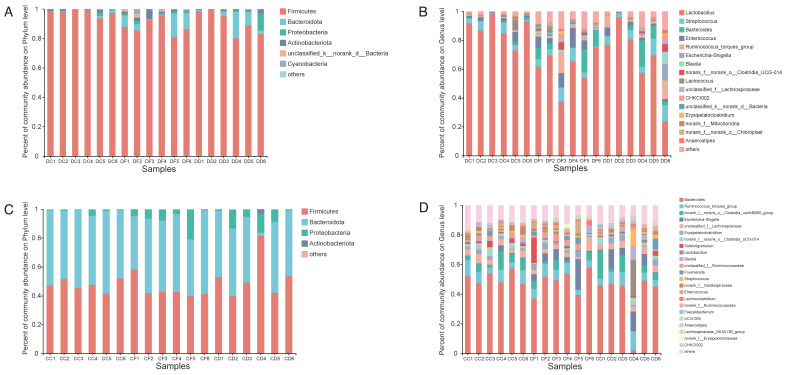
The difference in distribution in the intestinal microbiota at the phylum (**A**,**C**) and genus (**B**,**D**) level. Histogram of species composition in the duodenum (**A**,**B**) and cecum (**C**,**D**). Species with an abundance of more than 0.02 are listed in each graph, and those with an abundance of less than 0.02 were assigned to “others”. DC, duodenal contents of chickens fed with non-supplemented diet and water; DD, duodenal contents of chickens fed with 0.5% KDF; DF, duodenal contents of chickens supplied with 0.1% KDF in drinking water; CC, cecum contents of chickens fed with non-supplemented diet and water; CD, cecum contents of chickens fed with 0.5% KDF; CF, cecum contents of chickens supplied with 0.1% KDF in drinking water.

In this study, dominant taxa were defined as taxa with total relative abundances greater than 0.5% at different taxonomic levels. After filtering the relative abundances lower than 0.5% in all groups, 10 and 7 phyla were identified in the duodenum and cecum microbiota, respectively. Both in the duodenum and cecum, Firmicutes, Bacteroidota and Proteobacteria were the most abundant phyla (Figure 4A,C). At the genus level, 175 and 252 taxa were identified in the duodenal and cecal samples, respectively. In the duodenum, Lactobacillus, Streptococcus, Bacteroides, Enterococcus and Ruminococcus_torques_group were the most abundant genus (Figure 4B), whereas a higher abundance of Bacteroides, Ruminococcus_torques_group, Escherichia-Shigella and unclassified_f_Lachnospiraceae was observed in the cecum (Figure 4D).

**Figure 5:**
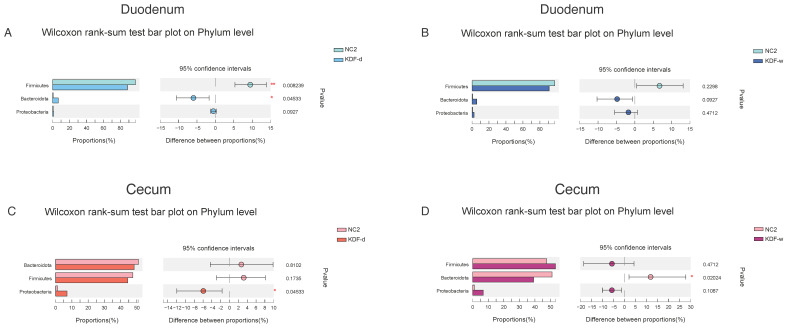
Changes in the intestinal microbiota of chickens in the investigated groups at the phylum level. (**A**) Duodenum contents in the NC2 group compared with those in the KDF-d group. (**B**) Duodenum contents in the NC2 group compared with those in the KDF-w group. (**C**) Cecum contents in the NC2 group compared with those in the KDF-d group. (**D**) Cecum contents in the NC2 group compared with those in the KDF-w group. NC2: chickens supplied with non-supplemented diet and water without infection (n = 6); KDF-d: chickens supplied with 0.5% KDF in diet without infection (n = 6); KDF-w: chickens supplied with 0.1% KDF in drinking water without infection (n = 6). * *p* < 0.05, ** *p* < 0.01.

**Figure 6:**
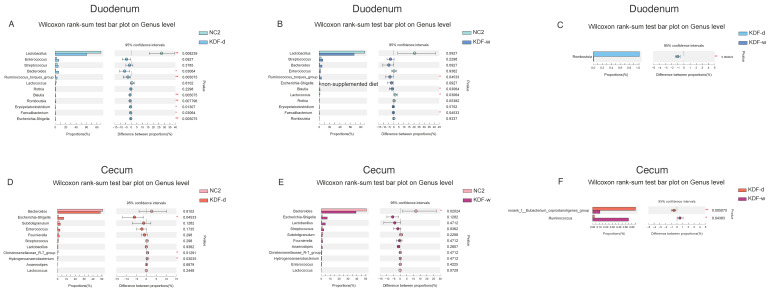
Changes in the intestinal microbiota of chickens in different groups at the genus level. (**A**) Duodenum contents in the NC2 group compared with those in the KDF-d group. (**B**) Duodenum contents in the NC2 group compared with those in the KDF-w group. (**C**) Duodenum contents in the KDF-d group compared with those in the KDF-w group. (**D**) Cecum contents in the NC2 group compared with those in the KDF-d group. (**E**) Cecum contents in the NC2 group compared with those in the KDF-w group. (**F**) Cecum contents in the KDF-d group compared with those in the KDF-w group. NC2: chickens supplied with non-supplemented diet and water without infection (n = 6); KDF-d: chickens supplied with 0.5% KDF in diet without infection (n = 6); KDF-w: chickens supplied with 0.1% KDF in drinking water without infection (n = 6). * *p* < 0.05, ** *p* < 0.01.

In the duodenum, compared with the NC2 group, the relative abundance of Firmicutes was decreased in the KDF treatment group, whereas Bacteroidota and Proteobacteria were increased (Figure 5A,B). The relative abundance of the genera Streptococcus, Bacteroides, Ruminococcus_torques_group, Blautia, Erysipelatoclostridium, Faecalibacterium and Escherichia-Shigella in the KDF treatment group were elevated, whereas Lactobacillus and Lactococcus were reduced (Figure 6A,B). The levels of Enterococcus and Romboutsia were increased in the KDF-d group but were decreased in the KDF-w group (Figure 6A,B). Notably, compared to the KDF-w group, the level of Romboutsia was significantly higher in the KDF-d group (*p* < 0.01) (Figure 6C).

In the cecum, compared with the NC2 group, the relative abundance of Bacteroidota was decreased in the KDF treatment group, whereas that of Proteobacteria was increased (Figure 5C,D). The relative abundance of Firmicutes was decreased in the KDF-d group but increased in the KDF-w group (Figure 5C,D). At the genus level, the proportions of Escherichia-Shigella and Fournierella in the KDF treatment group were increased (Figure 6D,E). In contrast, the relative abundances of Bacteroides, Christensenellaceae_R-7_group, Hydrogenoanaerobacterium and Lactococcus were reduced (Figure 6D,E). However, the relative abundances of Subdoligranulum and Enterococcus were higher in the KDF-d group and lower in the KDF-w group (Figure 6D,E). Conversely, the relative abundances of Streptococcus, Lactobacillus and Anaerostipes were decreased in the KDF-d group but increased in the KDF-w group (Figure 6D,E). Compared to the KDF-w group, the abundance of norank_f__Eubacterium_coprostanoligenes_group was significantly higher in the KDF-d group (*p* < 0.01), whereas that of Ruminococcus was significantly lower (*p* < 0.05) (Figure 6F).

## 3. Discussion

As an antibiotics substitute, KDF has shown promising effects in enhancing animal growth performance, resistance to intestinal disease and maintenance of gastrointestinal homeostasis [24,25]. However, few studies have provided direct evidence of the preventive effect of KDF with respect to infectious disease. In the present study, a chicken model of *S. pullorum* infection, an important poultry pathogen, was selected to evaluate the effectiveness of KDF in preventing intestinal pathogenic bacteria infection. According to the results, supplementation of KDF in both feed and drinking water reduced the loads of *S. pullorum* and alleviated the intestinal pathological changes in the cecum after infection, indicating the effective prevention of KDF against *S. pullorum* infection. Furthermore, the pH of the GIT was significantly decreased by supplementation of KDF. Organic acid mainly works by creating a low-pH environment. For example, in a previous study, FA supplementation in broiler diet significantly decreased the pH value and colonization of *S. gallinarum* in the digestive tract [26]. Dietary FA significantly reduced the GIT pH and improved performance of broiler chickens [26]. A lower pH in the GIT can be beneficial in several ways, for example, by increasing the activity of the enzyme pepsin, which enhances utilization of protein; increasing the digestibility of nutrients by improving intestinal morphology; and possibly reducing pathogenic bacteria, decreasing viabilities at low pH and selecting acid-resistant probiotics [10,27]. In this study, pH environments mimicking that in the GIT were created by dissolving KDF in vitro, which significantly inhibited the survival of *S. pullorum*. Therefore, the acid environment caused by KDF could be responsible for its prevention of *S. pullorum* infection in vivo.

Microbial diversity and abundance in the gut significantly affect host health, with many essential functions, such as assistance in food digestion, modulation of immune responses and participation in the development of gut epithelial cells [28,29]. Diet and feed additives are common factors that affect the host intestinal flora. Although the effect of KDF on one pathogenic bacterium in vitro and in vivo was confirmed in this study, the possible effects of KDF on changes in the microbial community in the gut are still unknown. Therefore, we further investigated the influence of KDF on the microorganisms in duodenum and cecum of chickens. The addition of 0.5% KDF to feed modified the diversity of duodenal and cecum microorganisms, which showed increased abundance but decreased diversity. The addition of 0.1% KDF to drinking water resulted in a similar trend to that observed in the 0.5% KDF feeding group with respect to the diversity of the intestinal flora but with no statistical significance, probably due to the greater discreteness of the data compared with that in feeding group.

**Figure 7:**
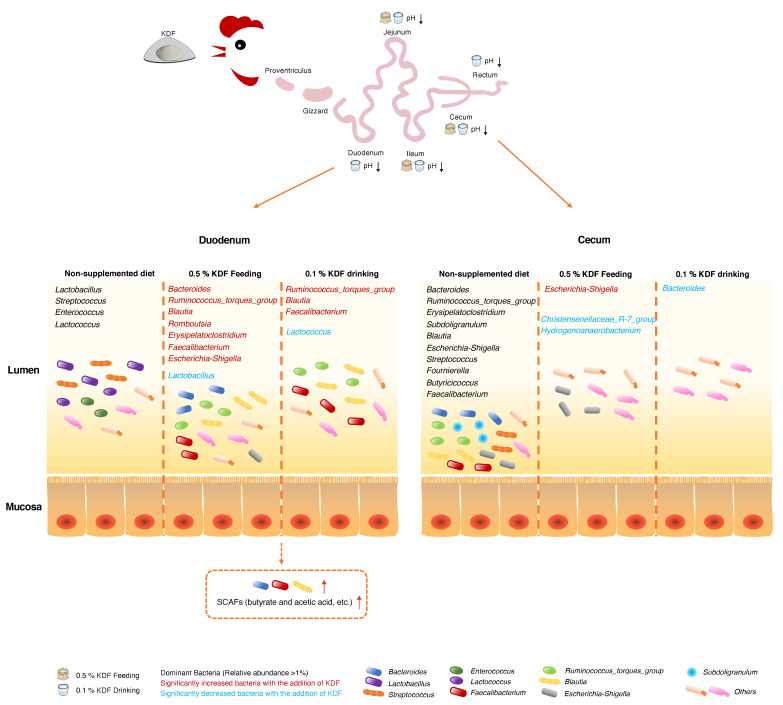
Schematic diagram of changes in pH and intestinal flora of chickens after KDF supplementation.

In the duodenum, the relative abundances of Bacteroides, Blautia, Ruminococcus_torques_group and Faecalibacterium were significantly higher in the KDF treatment groups than in other group. As the most prevalent and abundant members of the intestinal microbiota, Bacteroides have long been recognized as commensal colonizers, playing roles in maintenance of intestinal homeostasis and resistance against enteric pathogens [30]. For example, Bacteroides can induce goblet cell differentiation and increase mucin gene expression and goblet cell number [31]. Bacteroides can compete with pathogens for host-derived amino acids and monosaccharides and produce commensal colonization factors to enhance the intestinal immune barrier, thereby protecting the host against pathogens [32,33]. Bacteroides also produce short-chain fatty acids (SCFAs) that can acidify the environment and slow down the growth of pathogens [34]. Blautia is a genus of anaerobic bacteria with probiotic characteristics. It has been confirmed to contribute to alleviation of inflammatory diseases and metabolic diseases [35]. It also exhibits antibacterial activity against specific microorganisms in the gut by producing bacteriocins [36]. Additionally, Blautia can affect the composition of intestinal microbiota and selectively inhibit the proliferation of *C. perfringens* and vancomycin-resistant enterococci [37]. Furthermore, all Blautia strains can use glucose and formate to produce acetic acid, succinic acid and lactic acid, playing an important role in maintaining environmental balance and preventing inflammation in the intestine, where acetic acid also participates in energy synthesis in most eukaryotic cells [36,38,39]. *Faecalibacterium prausnitzii* are promising candidates as next-generation probiotics. *F. prausnitzii* is an important producer of butyrate, which is the main energy source of intestinal epithelial cells of the large intestine and is considered to be effective in promoting epithelial growth [40,41]. *F. prausnitzii* also exhibit anti-inflammatory function. The MAM protein found in *F. prausnitzii* supernatant can block NF-κB activation and the production of proinflammatory cytokine IL-8 [42]. *F. prausnitzii* directly produces bioactive anti-inflammatory molecules, such as shikimic and salicylic acids [43]. In addition, *F. prausnitzii* has been found to be a strong inducer of regulatory T cells secreting IL-10 [44]. Ruminococcus_torques_group has been found to be closely associated with obesity and metabolic syndrome and benefits metabolism by increasing production of deoxycholic acid and ameliorating obesity via the bile-acid–adipose TGR5 axis [45,46]. Following supplementation of KDF in chickens, the significant increase in levels of the above-mentioned commensal colonizers or bacteria with probiotic characters may play critical roles in intestinal homeostasis by maintaining the intestinal barrier, modulating inflammation, providing energy for intestinal cells and resisting pathogen infection (Figure 7).

The relative abundances of Erysipelatoclostridium and Romboutsia were also significantly higher in the KDF-treated group relative to those in other investigated groups. Romboutsia has been reported to be associated with obesity in humans and is positively correlated with high-density lipoprotein cholesterol and superoxide dismutase levels [47]. Erysipelatoclostridium is considered an opportunistic pathogen in the human gut. It has been found in high abundance in the feces of gout patients and is correlated with hepatic fat content in humans [48,49]. In animals, Erysipelatoclostridium may be key microbial markers that can predict early-life diarrhea in neonatal calves [50]. In addition, Erysipelatoclostridium has been reported to be highly abundant in the hindgut digesta of healthy calves [51]. However, the functions of Erysipelatoclostridium in poultry have not been reported. The outcomes of changes in these two species require further investigations.

Notably, the relative abundances of Lactobacillus and Lactococcus in the duodenum decreased after KDF treatment. Low pH may cause a reduction in lactic-acid-producing bacteria (LAB) in the duodenum. A study revealed that supplementation of FA in the diet of piglets reduced the abundance of Lactobacilli in the intestine [52]. Dittoe et al. suggested that the most significant challenge with respect to the application of organic acid feed additives is their potentially detrimental effect on LAB [53]. Another challenge identified in this study is the elevation of Escherichia-Shigella abundance. Enterohemorrhagic Escherichia coli (EHEC) can sense Bacteroides spp. as landmark organisms to find their infection niche along the gastrointestinal tract [54]. In an inflamed gut, SCFAs of Bacteroides may be exploited by facultative anaerobic pathogens as a carbon source for anaerobic respiration [55,56]. Additionally, low pH and high concentrations of weak organic acids in the GIT can select undesirable, acid-tolerant microorganisms. For example, pathogenic E. coli showed enhanced resistance to extreme acidic conditions after exposure to SCFAs [57]. Thus, the possible evolution of facultative bacteria in the intestine in response to local environment changes induced by organic acid merits attention.

In the present study, the situations observed in the cecum were quite different from those in the duodenum, with changes only in Bacteroides, Christensenellaceae_R-7_group, Hydrogenoanaerobacterium and Escherichia-Shigella. Several studies have revealed that organic acid administered in feed or water are mainly metabolized and absorbed in the upper gastrointestinal segments of poultry, which is consistent with the pH changes detected after KDF treatment in the present study. Therefore, the minor influence on the microbiota in cecum probably results from the weaker effect of KDF on the distal intestine [58,59].

## 4. Materials and Methods

### 4.1. Bacterial Strains and Culture Conditions

*S. pullorum* C79-3 was obtained from the China Institute of Veterinary Drug Control. The strain was aerobically cultured in tryptic soy broth (TSB) or tryptic soytone agar (TSA) at 37 °C.

### 4.2. Animal Experimental Design and Sample Collection

Chickens were hatched from SPF White Leghorn chicken eggs for this study. Eggs from SPF White Leghorn chickens were obtained from Boehringer Ingelheim Vital Biotechnology Co, Ltd. (Beijing, China) and were hatched at 37 °C in an incubator. The purity of KDF added into feed and drinking water was 95% and 98%, respectively. KDF was purchased from Alliance Biotech Co., Ltd. (Sanming, Fujian, China). All chickens were raised in SPF chicken isolators and provided with feed and water ad libitum.

The animal experimental design is shown in Figure 1. For the infection experiment (Figure 1A), a total of 96 1-day-old SPF Leghorn chickens were randomly assigned into 4 groups: Group 1: chickens supplied with 0.5% KDF in diet infected with *S. pullorum* (n = 30), indicated as KDF-d (SP); Group 2: chickens supplied with 0.1% KDF in drinking water infected with *S. pullorum* (n = 30), indicated as KDF-w (SP); Group 3: chickens supplied with non-supplemented diet and water infected with *S. pullorum* (n = 30), indicated as CSP; Group 4: chickens supplied with non-supplemented diet and water without infection (n = 6), indicated NC1.

Following two days of KDF pretreatment at an age of one day, all chickens in the infected groups were inoculated with the *S. pullorum* C79-3 at a dose of 5 × 10^8^ CFU per chicken via gastric gavage (Figure 1A). Changes in the body weight of the chickens, the bacterial loads of the cecum, the pathological changes of the cecum and the pH of the GIT were then recorded at various time point post infection.

At 1, 3, 5 and 7 dpi, six chickens were randomly selected from groups 1–3 and euthanized for detection of bacterial loads in one cecum. The cecum tissue was collected and homogenized. Plate counts were used to determine the number of viable bacterial numbers in the homogenized tissue. The other cecum of the chickens from at 3, and 7 dpi was used for observation of cecum morphology (n = 3) and histopathological analysis (n = 3), respectively. Three chickens were randomly selected from group 4 at 3 and 7 dpi, respectively, and euthanized for morphology and histopathological analysis of the cecum as negative control. For histopathological analysis, cecum tissues were fixed in 4% paraformaldehyde phosphate buffer solution for 48 h at room temperature. Samples were embedded in paraffin and sectioned at 5 μm for HE staining. The cecum lesions were observed under a microscope. At 9 dpi, the remaining chickens from groups 1–3 (six in each group) were euthanized for detection of bacterial loads in the cecum and analysis of the pH conditions of different sections of the gut. The proventriculus, gizzard, duodenum, jejunum, ileum, cecum and rectum were collected, and the pH values of the contents in different sections were determined by a digital pH meter.

For intestinal microbiota analysis (Figure 1B), a total of 18 chickens were divided into 3 groups without infection: Group 5: chickens supplied with 0.5% KDF in diet without infection (n = 6), indicated as KDF-d; Group 6: chickens supplied with 0.1% KDF in drinking water without infection (n = 6), indicated as KDF-w; and Group 7: chickens supplied with non-supplemented diet and water without infection (n = 6), indicated as NC2. Twenty-one days after KDF supplementation at 1 day old, all chickens were euthanized, and the luminal contents of the duodenum and cecum were collected using sterile forceps. The samples were snap-frozen in liquid nitrogen and stored at −80 °C for analysis of the intestinal microbiota.

### 4.3. Determination of Antibacterial Activity of KDF In Vitro

The pH (3.02, 5.15 and 5.81) of different parts of the GIT of chickens were simulated in vitro by dissolving different concentrations of KDF in ddH_2_O. The survival of *S. pullorum* C79-3 was detected. Overnight cultures of bacterial strains were subcultured at 1:100 into fresh media and grown to mid-log phase, then diluted to 1 × 10^8^ CFU/mL. A volume of 1 mL of the bacterial solution was centrifuged at 5000 rpm for 5 min, and supernatants were discarded. Bacteria were washed and resuspended in 1mL of KDF solution with varying pH values (3.02, 5.15 and 5.81) and in saline as a control. The agar plates were incubated at 37 °C for 12 h, and bacterial counts were quantified.

### 4.4. DNA Extraction and 16S rRNA Gene Sequencing

Total microbial genomic DNA was extracted from samples of duodenal and cecal contents of uninfected chickens, including the KDF-d, KDF-w and NC2 groups, using a TIANamp stool DNA kit (TIAN GEN) according to the manufacturer’s instructions. The quality and concentration of DNA were determined by 1.0% agarose gel electrophoresis and a NanoDrop^®^ ND-2000 spectrophotometer (Thermo Scientific Inc., Waltham, MA, USA).

The hypervariable regions (V3-V4) of the bacterial 16S rRNA coding gene were amplified with primer pairs 341 F (5′-CCTAYGGGRBGCASCAG-3′) and 806 R (5′-GGACTACNNGGGTATCTAAT-3′) by an ABI GeneAmp^®^ 9700 PCR thermocycler (ABI, Carlsbad, CA, USA). All samples were amplified in triplicate. The PCR products were extracted from 2% agarose gel and purified using an AxyPrep DNA gel extraction kit (Axygen Biosciences, Union City, CA, USA) according to the manufacturer’s instructions and quantified using a Quantus™ fluorometer (Promega, Madison, WI, USA). Purified amplicons were pooled in equimolar amounts and paired-end-sequenced on a NovaSeq PE250 platform (Illumina, San Diego, CA, USA) by Majorbio Bio-Pharm Technology Co. Ltd. (Shanghai, China) according to standard protocols.

### 4.5. Bioinformatics Analysis

The optimized sequences after quality control were spliced and clustered into operational taxonomic units (OTUs) using UPARSE 7.1 with a 97% sequence similarity level. The most abundant sequence for each OTU was selected as a representative sequence. The taxonomy of each OTU representative sequence was analyzed by RDP Classifier version 2.2 against the 16S rRNA gene database (e.g., Silva v138) using a confidence threshold of 0.7.

Alpha diversity (Chao and Simpson indices) was determined to analyze the microbial diversity in the environment. Differences in alpha diversity between groups were tested using the Wilcoxon rank sum test. The similarity among the microbial communities in different samples was determined by beta diversity and analyzed using PCoA based on Bray–Curtis dissimilarity. A PERMANOVA test was used to assess the percentage of variation explained by the treatment, along with its statistical significance.

### 4.6. Statistical Analysis

Statistical analyses between two groups were performed with GraphPad Prism 8 (GraphPad Software, La Jolla, CA, USA) using a two-tailed Student’s *t*-test unless otherwise indicated, with results expressed as means ± standard deviations (SD). * *p* < 0.05, ** *p* < 0.01, *** *p* < 0.001, **** *p* < 0.0001.

## 5. Conclusions

In conclusion, these results of the present study demonstrate that KDF reduced the bacterial loads and the degree of pathological changes in the cecum, improved digestion and reduced the pH of the GIT of chickens infected with *S. pullorum*. These data provide evidence for the clinical application of KDF in the prevention of enteropathogenic bacterial infections in chickens. Additionally, KDF changed the diversity of the chicken intestinal microflora, with a significant increase in the abundance of several beneficial bacteria. The functions of the changed bacterial species after supplementation with KDF in feed and drinking water with respect to maintenance of intestinal homeostasis and possible risk merit further investigations.

## Figures and Tables

**Table 1:** The pH values in different GIT segments of inoculated chickens receiving no KDF or KDF supplementation in diet or water.

GIT Segment	CSP	KDF-d (SP)	KDF-w (SP)
Proventriculus	4.91 ± 0.23 ^a^	4.91 ± 0.14 ^a^	4.84 ± 0.24 ^a^
Gizzard	4.02 ± 0.15 ^a^	3.93 ± 0.15 ^a^	3.02 ± 0.14 ^b^
Duodenum	6.05 ± 0.16 ^a^	5.75 ± 0.36 ^b^	5.15 ± 0.17 ^c^
Jejunum	6.46 ± 0.24 ^a^	5.56 ± 0.22 ^b^	5.00 ± 0.19 ^c^
Ileum	6.34 ± 0.24 ^a^	5.58 ± 0.26 ^b^	5.21 ± 0.26 ^c^
Cecum	6.15 ± 0.10 ^a^	5.87 ± 0.12 ^b^	5.81 ± 0.13 ^b^
Rectum	5.75 ± 0.13 ^a^	5.66 ± 0.15 ^a^	5.27 ± 0.09 ^b^

CSP: chickens supplied with non-supplemented diet and water infected with *S. pullorum* (n = 6); KDF-d (SP): chickens supplied with 0.5% KDF in diet infected with *S. pullorum* (n = 6); KDF-w (SP): chickens supplied with 0.1% KDF in drinking water infected with *S. pullorum* (n = 6). Significant differences in the values are marked with different characters. No significant differences in the values are marked with the same character.

**Table 2:** PERMANOVA analysis based on Bray–Curtis dissimilarities.

Pairwise Comparison	Sum of Squares	Mean Square	F. Model	R^2^	*p*.Value	*p*.Adjust
Duodenum						
NC2 vs. KDF-d	0.22114	0.22114	2.65893	0.21004	0.009 **	0.009
NC2 vs. KDF-w	0.14303	0.14303	1.3527	0.11915	0.232	0.232
KDF-d vs. KDF-w	0.08288	0.08288	0.76399	0.07098	0.67	0.67
Cecum						
NC2 vs. KDF-d	0.20937	0.20937	2.26304	0.18454	0.022 *	0.022
NC2 vs. KDF-w	0.17491	0.17491	1.43479	0.12548	0.13	0.13
KDF-d vs. KDF-w	0.31297	0.31297	2.23351	0.18257	0.019 *	0.019

NC2: chickens supplied with non-supplemented diet and water without infection (n = 6); KDF-d: chickens supplied with 0.5% KDF in diet without infection (n = 6); KDF-w: chickens supplied with 0.1% KDF in drinking water without infection (n = 6). Data of each group were obtained from chickens at 21 days of age (n = 6). * *p* < 0.05, ** *p* < 0.01.

## Data Availability

The raw reads were deposited into the NCBI Sequence Read Archive (SRA) database (accession number PRJNA856798).

## Supplementary Materials

The following supporting information can be downloaded at: https://www.mdpi.com/article/10.3390/antibiotics11091265/s1. Figure S1: Changes in body weight of the chickens; Figure S2: Venn diagram analysis of species based on OTU levels in intestinal flora of the duodenum; Figure S3: Venn diagram analysis of species based on OTU levels in intestinal flora of the cecum; Table S1: Valid sequence of each sample.

## Abbreviations

dpi	Days post infection
FA	Formic acid
GIT	Gastrointestinal tract
KDF	Potassium diformate
LAB	Lactic-acid-producing bacteria
MIC	Minimum inhibitory concentration
PCoA	Principal coordinate analysis
PERMANOVA	Permutational multivariate analysis of variance
SCFAs	Short-chain fatty acids
TSB	Tryptic soy broth
