# Supplementary material for: The Feed Additive Potassium Diformate Prevents Salmonella enterica Serovar Pullorum Infection and Affects Intestinal Flora in Chickens
Source: Antibiotics (Basel). 2022 Sep 18;11(9):1265. doi: 10.3390/antibiotics11091265 (PMC9495629; doi:10.3390/antibiotics11091265)
Supplement: Supplementary file 1 [file antibiotics-11-01265-s001.zip › antibiotics-1829046-supplementary.pdf]

Supplementary Materials

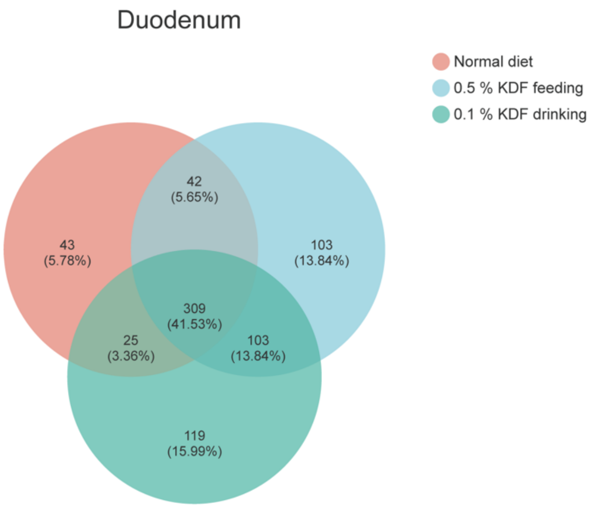

Figure S1. Venn diagram analysis of species based on OTU levels in intestinal flora of duodenum.

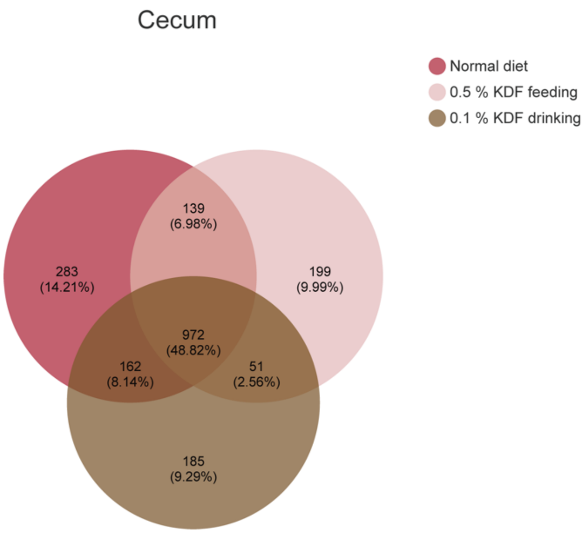

Figure S2. Venn diagram analysis of species based on OTU levels in intestinal flora of Cecum.

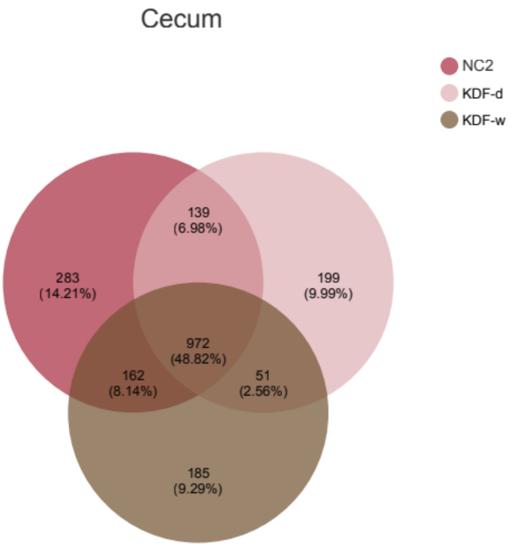

Figure S3. Venn diagram analysis of species based on OTU levels in intestinal flora of the cecum.

Table S1. Valid sequence numbers of each sample.

Intestinal segment	Sample Name	Sequence number
	Normal diet	
	DC1	109958
	DC2	85932
	DC3	106668
	DC4	100132
	DC5	60069
	DC6	111345
	0.5 % KDF feeding	
	DF1	88380
	DF2	89947
**Duodenum**	DF3	119747
	DF4	83262
	DF5	73058
	DF6	73724
	0.1 % KDF drinking	
	DD1	98179
	DD2	73807
	DD3	112416
	DD4	148898
	DD5	64194
	DD6	84507
	Normal diet	
	CC1	116910
	CC2	122786
	CC3	117775
	CC4	84196
	CC5	83389
	CC6	84793
	0.5 % KDF feeding	
	CF1	83866
	CF2	198371
**Cecum**	CF3	159898
	CF4	151808
	CF5	135463
	CF6	85856
	0.1 % KDF drinking	
	CD1	115935
	CD2	62077
	CD3	60111
	CD4	32748
	CD5	120133
	CD6	68676

DC, Duodenal contents of normally fed chickens; DD, Duodenal contents of chickens fed with 0.5% KDF; DF, Duodenal contents of chickens supplied with 0.1% KDF in drinking water; CC, Cecum contents of normally fed chickens; CD, Cecum contents of chickens fed with 0.5% KDF; CF, Cecum contents of chickens supplied with 0.1% KDF in drinking water.
